# Supplementary material for: Obesity-Associated NAFLD Coexists with a Chronic Inflammatory Kidney Condition That Is Partially Mitigated by Short-Term Oral Metformin
Source: Nutrients. 2025 Jun 26;17(13):2115. doi: 10.3390/nu17132115 (PMC12251041; doi:10.3390/nu17132115)
Supplement: Supplementary file 1 [file nutrients-17-02115-s001.zip › nutrients-3687454-supplementary.pdf]

Supplemental Figure S1. Proteins levels of GST-P, Cu/ZnSOD, and MnSOD in kidneys of lean rats fed with AIN-93 G diet with or without metformin.

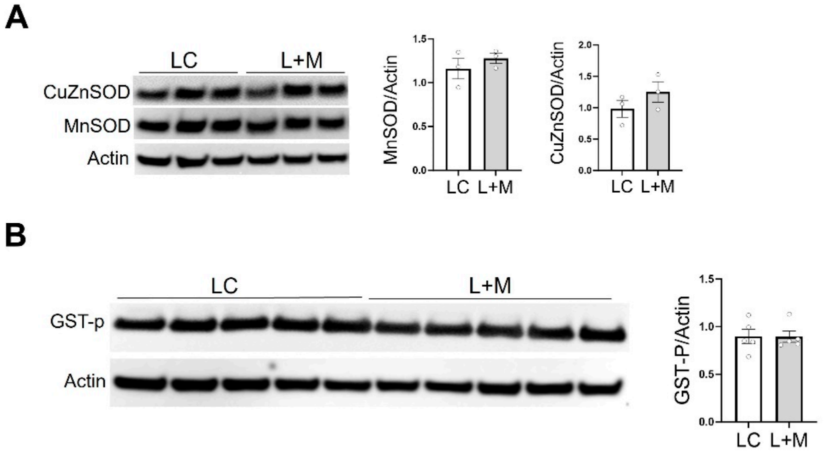

Kidneys from lean rats fed with AIN-93 G diet with or without metformin (1 g/kg) ad libitum access were isolated and homogenized in phosphate buffer followed by preparation of RIPA lysates. (A) Twenty ug of proteins were resolved on a SDS-PAGE followed by western blotting with primary antibodies as indicated. Detection was performed with secondary antibody conjugated with horse radish peroxidase enzyme and chemiluminescence reagent. (A) Representative Western blot images of copper zinc superoxide dismutase (CuZnSOD) and Manganese superoxide dismutase (MnSOD) proteins in renal lysates from control (LC) or metformin-treated lean rats (L+M). β-actin was used as a loading control. Graphs showing densitometry analysis of protein bands of superoxide dismutase I (CuZnSOD) and superoxide dismutase II (MnSOD) proteins normalized to the corresponding β-actin band. Data are presented as mean ± SEM (n = 3 per group). (B) Representative Western blot image of GST-P protein in renal lysates from control (LC) or metformin-treated lean rats (L+M). β-actin was used as a loading control. The graph showing densitometry analysis of protein bands of GST-P protein normalized to the corresponding β-actin band. Data are presented as mean ± SEM (n = 5 per group). Statistical significance was determined by a non-parametric Mann Whittney U test for both (A) and (B).

Supplemental Figure S2. Total and cleaved caspase-3 protein levels in kidneys of lean rats fed with AIN-93 G diet with or without metformin.

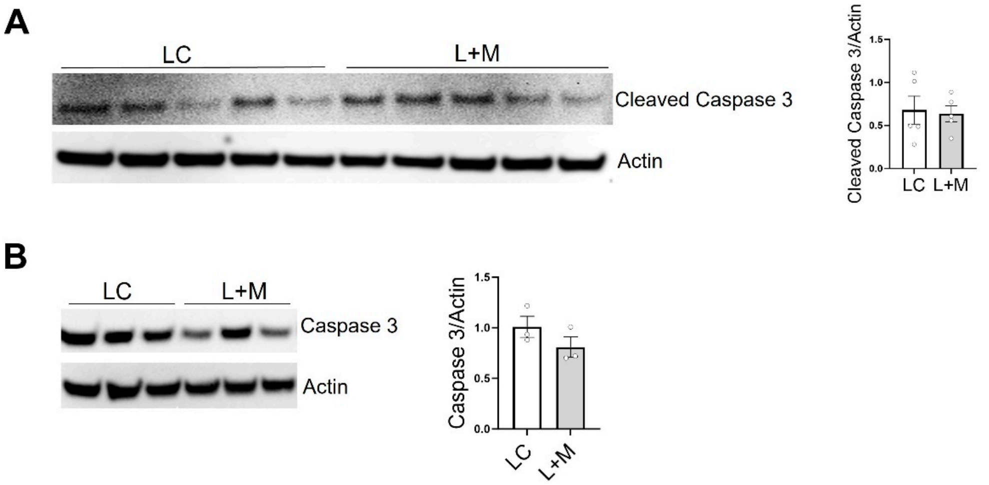

Kidneys from lean rats fed with AIN-93 G diet with or without metformin (1 g/kg) ad libitum access were isolated and homogenized in phosphate buffer followed by preparation of RIPA lysates. (A) Twenty ug of proteins were resolved on a SDS-PAGE followed by western blotting with primary antibodies as indicated. Detection was performed with secondary antibody conjugated with horse radish peroxidase enzyme and chemiluminescence reagent. (A) Representative Western blot image of cleaved caspase-3 protein in renal lysates from control (LC) or metformin-treated lean rats (L+M). β-actin was used as a loading control. The graph showing densitometry analysis of protein bands of cleaved caspase-3 protein normalized to the corresponding β-actin band. Data are presented as mean ± SEM (n = 5 per group). (B) Representative Western blot image of caspase-3 protein in renal lysates from control (LC) or metformin-treated lean rats (L+M). β-actin was used as a loading control. The graph showing densitometry analysis of protein bands of caspase-3 protein normalized to the corresponding β-actin band. Data are presented as mean ± SEM (n = 3 per group). Statistical significance was determined by a non-parametric Mann Whittney U test for both (A) and (B).
